# Supplementary material for: HIVprotI: an integrated web based platform for prediction and design of HIV proteins inhibitors
Source: J Cheminform. 2018 Mar 9;10:12. doi: 10.1186/s13321-018-0266-y (PMC5845081; doi:10.1186/s13321-018-0266-y)
Supplement: Supplementary file 2 — Additional file 2. Source code of HIVProtI web server. [file 13321_2018_266_MOESM2_ESM.zip › HIVprotI_Source-code/hivprotI/weka-3-6-6/doc/weka/classifiers/mi/supportVector/MIPolyKernel.html]

MIPolyKernel


---


|  |  |  |
| --- | --- | --- |
| **Overview** \| **Package** \| **Class** \| **Tree** \| **Deprecated** \| **Index** \| **Help** \| **Weka's home** | |  |
| PREV CLASS   **NEXT CLASS** | **FRAMES**    **NO FRAMES**     **All Classes** |  |
| SUMMARY: NESTED \| FIELD \| CONSTR \| METHOD | DETAIL: FIELD \| CONSTR \| METHOD |  |


---


## weka.classifiers.mi.supportVector Class MIPolyKernel

```
java.lang.Object
  weka.classifiers.functions.supportVector.Kernel
      weka.classifiers.functions.supportVector.CachedKernel
          weka.classifiers.functions.supportVector.PolyKernel
              weka.classifiers.mi.supportVector.MIPolyKernel
```

**All Implemented Interfaces:**: java.io.Serializable, CapabilitiesHandler, MultiInstanceCapabilitiesHandler, OptionHandler, RevisionHandler

---

``` public class MIPolyKernel extends PolyKernel implements MultiInstanceCapabilitiesHandler ```

The polynomial kernel : K(x, y) = <x, y>^p or K(x, y) = (<x, y>+1)^p


Valid options are:

```
 -D
  Enables debugging output (if available) to be printed.
  (default: off)
```

```
 -no-checks
  Turns off all checks - use with caution!
  (default: checks on)
```

```
 -C <num>
  The size of the cache (a prime number), 0 for full cache and 
  -1 to turn it off.
  (default: 250007)
```

```
 -E <num>
  The Exponent to use.
  (default: 1.0)
```

```
 -L
  Use lower-order terms.
  (default: no)
```

**Version:**
:   $Revision: 1.4 $

**Author:**
:   Eibe Frank (eibe@cs.waikato.ac.nz), Shane Legg (shane@intelligenesis.net) (sparse vector code), Stuart Inglis (stuart@reeltwo.com) (sparse vector code), Lin Dong (ld21@cs.waikato.ac.nz) (MIkernel)

**See Also:**: Serialized Form

---

**Constructor Summary**

`MIPolyKernel()`
  
          default constructor - does nothing.

`MIPolyKernel(Instances data,
int cacheSize,
double exponent,
boolean lowerOrder)`
  
          Creates a new `MIPolyKernel` instance.


| **Method Summary** | |
| --- | --- |
| `Capabilities` | `getCapabilities()`             Returns the Capabilities of this kernel. |
| `Capabilities` | `getMultiInstanceCapabilities()`             Returns the capabilities of this multi-instance kernel for the relational data. |
| `java.lang.String` | `getRevision()`             Returns the revision string. |

**Methods inherited from class weka.classifiers.functions.supportVector.PolyKernel**

`exponentTipText, getExponent, getOptions, getUseLowerOrder, globalInfo, listOptions, setExponent, setOptions, setUseLowerOrder, toString, useLowerOrderTipText`

**Methods inherited from class weka.classifiers.functions.supportVector.CachedKernel**

`buildKernel, cacheSizeTipText, clean, eval, getCacheSize, numCacheHits, numEvals, setCacheSize`

**Methods inherited from class weka.classifiers.functions.supportVector.Kernel**

`checksTurnedOffTipText, debugTipText, forName, getChecksTurnedOff, getDebug, makeCopies, makeCopy, setChecksTurnedOff, setDebug`

**Methods inherited from class java.lang.Object**

`equals, getClass, hashCode, notify, notifyAll, wait, wait, wait`

**Constructor Detail**

### MIPolyKernel

```
public MIPolyKernel()
```

:   default constructor - does nothing.

---


### MIPolyKernel

```
public MIPolyKernel(Instances data,
                    int cacheSize,
                    double exponent,
                    boolean lowerOrder)
             throws java.lang.Exception
```

:   Creates a new `MIPolyKernel` instance.

    **Parameters:**: `data` - the training dataset used.: `cacheSize` - the size of the cache (a prime number): `exponent` - the exponent to use: `lowerOrder` - whether to use lower-order terms **Throws:**: `java.lang.Exception` - if something goes wrong


**Method Detail**

### getCapabilities

```
public Capabilities getCapabilities()
```

:   Returns the Capabilities of this kernel.

    :   **Specified by:**: `getCapabilities` in interface `CapabilitiesHandler` **Overrides:**: `getCapabilities` in class `PolyKernel`
    :   **Returns:**: the capabilities of this object **See Also:**: `Capabilities`

---


### getMultiInstanceCapabilities

```
public Capabilities getMultiInstanceCapabilities()
```

:   Returns the capabilities of this multi-instance kernel for the
    relational data.

    :   **Specified by:**: `getMultiInstanceCapabilities` in interface `MultiInstanceCapabilitiesHandler`
    :   **Returns:**: the capabilities of this object **See Also:**: `Capabilities`

---


### getRevision

```
public java.lang.String getRevision()
```

:   Returns the revision string.

    :   **Specified by:**: `getRevision` in interface `RevisionHandler` **Overrides:**: `getRevision` in class `PolyKernel`
    :   **Returns:**: the revision


---


|  |  |  |
| --- | --- | --- |
| **Overview** \| **Package** \| **Class** \| **Tree** \| **Deprecated** \| **Index** \| **Help** \| **Weka's home** | |  |
| PREV CLASS   **NEXT CLASS** | **FRAMES**    **NO FRAMES**     **All Classes** |  |
| SUMMARY: NESTED \| FIELD \| CONSTR \| METHOD | DETAIL: FIELD \| CONSTR \| METHOD |  |


---
